# Assessing the monthly performance of daily precipitation products over Southeast Asia using the gauge-based analysis

**DOI:** 10.1371/journal.pone.0319477

**Published:** 2025-03-25

**Authors:** Yuxuan Xie, Jingyu Wang, Xia Wan, Yang Lyu

**Affiliations:** 1 National Institute of Education, Nanyang Technological University, Nanyang, Singapore; 2 Hubei Key Laboratory for Heavy Rain Monitoring and Warning Research, Institute of Heavy Rain, China Meteorological Administration (CMA), Wuhan, China; 3 Key Laboratory of Meteorology Disaster, Ministry of Education (KLME), Joint International Research Laboratory of Climate and Environment Change (ILCEC), Collaborative Innovation Center on Forecast and Evaluation of Meteorological Disasters (CIC-FEMD), Nanjing University of Information Science and Technology, Nanjing, China; Universiti Sains Malaysia, MALAYSIA

## Abstract

Accurate precipitation data is essential for understanding land surface processes and the hydrological cycle, particularly in Southeast Asia (SEA), where precipitation patterns are influenced by complex climatic interactions. This study evaluates the monthly performance of five widely-used daily precipitation products—CPC, CHIRPS, IMERG, ERA5, and PERSIANN—against the benchmark SAOBS dataset over SEA from January 2001 to December 2017. By aggregating daily data into monthly values, we identify the strengths and weaknesses of each product in capturing the spatial and temporal characteristics of precipitation in the tropical region. The evaluation includes analyses of data population, spatial distribution, and temporal variability at a monthly scale. Our findings reveal significant spatial heterogeneity in the performance of these products, emphasizing the importance of scale-specific assessments before their application in regional studies and management practices. Results indicate that the CPC product generally provides the most accurate monthly estimates, with the highest correlation coefficients and lowest root-mean-square errors. However, all products exhibit systematic biases, such as overestimation in high-rainfall regions and seasonal discrepancies. These findings underscore the need for regional calibration to improve the applicability of precipitation products for monthly-scale climate studies and resource management in SEA.

## 1. Introduction

Precipitation plays a vital role in land surface processes and the hydrological cycle, which has a significant impact on the water resources, industrial and agricultural production [1,2]. Spanning temporal scales from minutes to years, monthly precipitation serves as a key input for various applications. In water resource management, it supports reservoir and dam operations by guiding storage and release decisions [3,4]. Similarly, in agriculture, monthly precipitation data is crucial for modelling crop yields and planning resource allocation [5,6], helping ensure sustainability and resilience in these sectors. Southeast Asia (SEA) is the world’s largest archipelago, surrounded by the warmest oceans of the globe. The strong atmospheric instability and abundant moisture supply make this region one of the most prolific basins for precipitation. Moreover, located in the deep tropics, SEA’s climate is heavily influenced by tropical monsoons. The alteration between Northeast Monsoon in December-February and Southwest Monsoon in June-August marks the distinction between wet and dry seasons, which plays a critical role in defining the spatiotemporal distribution and intensity of rainfall across SEA [7,8].

The intricate land-sea interaction, monsoon variability, and diverse terrain of SEA collectively define its distinctive spatiotemporal precipitation patterns, which necessitate precise depiction to effectively support various endeavors such as agricultural planning and water resource management. Out of a wide range of precipitation data available, rain gauges have been historically trusted for measuring rainfall because of the accuracy. However, the gauge data may not adequately capture the full spatial variability of rainfall due to their scarce distribution. This issue is particularly pronounced in SEA, where certain terrain types are underrepresented. In recent years, a wide variety of precipitation products have been developed worldwide to address the observational gaps left by sparse gauge stations, and these products have been widely used for various research studies and management practices. As demonstrated by comparative evaluation studies across various regions, there exists notable spatial heterogeneity in the performance of these precipitation products [9–12]. Therefore, it is crucial to thoroughly evaluate these precipitation products to identify their strengths and weaknesses in accurately depicting precipitation patterns before applying them regionally. Although such evaluation studies have been conducted in various subdomains of SEA [13–16], a holistic assessment over the broad SEA is still lacking.

Regardless of the algorithms, all precipitation products are primarily derived from a combination of rain gauge observations, satellite estimates, and modeling results, with variations depending on the weighting of these data sources. For instance, the Integrated Multi-satellitE Retrievals for GPM (IMERG) integrates, merges, and inter-calibrates various infrared, microwave, and gauge observations to deliver continuous and quasi-global (65° N-S) precipitation measurements [17]. Although gauge observations are included, they are mainly used for regionalization and bias correction of satellite estimates [18], thus IMERG is often classified as a satellite-based product. In contrast, the National Oceanic and Atmospheric Administration (NOAA) Climate Prediction Center (CPC) precipitation product relies more heavily on surface station observations. The CPC gathers and analyzes data from a global network of ground-based weather stations to produce various precipitation products, with satellite data serving as a complement [19]. Unlike these observation-based methods, reanalysis precipitation data are generated through a consistent data assimilation system and model that integrates observations and a background model forecast to produce uniform gridded data [11,20]. Therefore, reanalysis products, such as the ECMWF Reanalysis v5 enhanced for the land component (ERA5-Land) [21], are typically considered modeling products.

For satellite-based products, the precipitation estimates are generated through unified algorithms, which have the advantage of more accessible satellite data and integration of various and high-resolution multi-satellite data. With the increasing training data and the advancement of the unified algorithm, the satellite-based products can produce increasingly accurate output for the climate studies. For the reanalysis-based product, it generates the reanalyzed precipitation estimates through a data assimilation system and model, which has the advantage of data consistency and comprehensive incorporation of the observation and the background model. Similar to the satellite-based products, the reanalysis-based product can produce the increasingly accurate output as well. with the advancement and improvement of the model and assimilation. For the gauge-based precipitation estimates products, it provides the unified analysis of the precipitation data, which has the advantage of high accuracy, and regional insights. With these different advantages, these different types of precipitation estimate products are widely used in climate research.

As previously mentioned, all existing global or quasi-global precipitation products are designed to cover the entire globe rather than specific subdomains. Consequently, they are not finely tuned for SEA, where the distinctive precipitation features, such as monsoon-driven rainfall variability and the complex interaction of local topography with regional climate patterns, require more localization and optimization. In this study, 3 satellite-based products (IMERG, Precipitation Estimation from Remotely Sensed Information using Artificial Neural Networks [PERSIANN], and Climate Hazards group Infrared Precipitation with Stations [CHIRPS]), 1 reanalysis product (ERA5-Land) and 1 gauge-based product (CPC) are examined against a local benchmark dataset to quantify their respective performance over SEA. This study is organized as follows: Section 2 introduces various precipitation products and the benchmark observation, along with the pre-processing procedures, evaluation metrics, and sub-region divisions. Section 3 presents a quantitative evaluation of the five precipitation products, focusing on their performance in terms of population density, spatial distribution, and seasonality across the entire SEA and its subdomains.

## 2. Materials and methodology

### 2.1. Precipitation products

#### A. CPC.

National Oceanic and Atmospheric Administration (NOAA) Climate Prediction Center (CPC) precipitation product [22] is a dataset based on the analysis of gauges to examine daily precipitation patterns across global land areas. Data from over 30,000 stations around the world, and over 17,000 stations for the “real-time version” data from 1979-2005. The data are obtained from various sources such as Global Telecommunication System (GTS), Cooperative Observer Program (COOP) and other national and international agencies. The daily analysis is conducted on a 0.125° grid globally and the product is released on a 0.5° grid worldwide, covering the period from 1979 to the present [23,24].

#### B. CHIRPS.

CHIRPS product incorporates interpolated gauge products and infrared Cold Cloud Duration (CDD) observations to estimate precipitation and provides daily precipitation data from 1981 to near present at 0.05° and 0.25° spatial resolution. CHIRPS covers a quasi-global area from 50°S to 50°N and all longitudes [25]. In this research, in order to align with the targeted resolution of benchmark dataset, the dataset with 0.25° resolution is analyzed.

#### C. ERA5.

ERA5 hourly data on single levels provides hourly estimates for an extensive array of atmospheric, land, and oceanic climate variables. The dataset spans the entire Earth from January 1940 to the present, represented on a 30km grid (0.25° resolution). The ERA5 precipitation data includes large scale rain rate, convective rain rate, mean total precipitation rate, snowfall rate water equivalent, etc. In this study, the subset of total precipitation data is used [26].

#### D. IMERG.

IMERG is the unified product derived from a network of partner satellites in the Global Precipitation Measurement (GPM) Mission [27]. The primary precipitation estimates in IMERG come from passive microwave (PMW) sensors, utilizing the Goddard Profiling Algorithm [28,29]. To fill in the gaps between PMW overpasses, a quasi-Lagrangian interpolation scheme (morphing) is applied to the gridded PMW precipitation estimates, using motion vectors derived from total precipitable water vapor from numerical models [30]. To ensure the accuracy of the data. IMERG uses a complex algorithm of intercalibration among output of putting raw data to PERSIANN and CPC algorithms. The IMERG precipitation data used in this study is the “Final Precipitation L3 Half Hourly 0.1° × 0.1° V06B” data ranging from 1 June 2000 to 30 September 2021 [30].

#### E. PERSIANN.

PERSIANN Precipitation Climate Data Record (PERSIANN-CDR) uses artificial neural network function procedures to provide daily rainfall estimates with a spatial resolution of 0.25° in the latitude from 60°S to 60°N and all longitudes. It covers the period from March 2000 to near present. The PERSIANN system uses geostationary infrared imagery and was subsequently expanded to incorporate both infrared and daytime visible imagery. This study uses the dataset of Version 1 and Revision 1 [31,32].

### 2.2. Benchmark observation and data pre-processing

For evaluation and analysis of all the precipitation products mentioned above. The daily gridded precipitation data SAOBS covering the period of January 1, 1981, to December 31, 2017, produced from the Southeast Aisa Climate Assessment and Dataset (SACA&D) are used in this study as the benchmark observation. The SAOBS dataset is a comprehensive collection of rainfall measurements specifically curated for the SEA region. This dataset integrates precipitation data from various meteorological stations across multiple countries within the region, which provides accurate, and long-term records of observed rainfall. By offering detailed insights into precipitation trends and anomalies, SAOBS is widely used in SEA climate studies and other industrial applications including agricultural planning and water resource management. SAOBS version 2 have choices of 0.5° × 0.5° and 0.25° × 0.25° resolution. In this research, aligning with the coarsest dataset to obtain a better result, 0.5° × 0.5° precipitation data is used. This dataset is currently one of the highest quality observational datasets for SEA and has been widely used to evaluate regional and global climate simulations because of its high accuracy control [33]. For example, in recent research of relationships between global warming and heat extremes in SEA, SAOBSv2.0 dataset is used as a benchmark data to verify all the daily maximum and daily minimum temperatures models [33]. Due to gaps in rain gauge coverage, particularly in Myanmar, Laos, and Cambodia, the absence of data for these regions in the SAOBS dataset may limit the comprehensiveness of the analysis, though addressing this impact is beyond the scope of this study.

The summary of all precipitation products is listed in Table 1. For fair comparison, all these datasets are all processed to uniform gridded datasets before further analysis. In this study, the python interface of the Earth System Modeling Framework (ESMFpy; https://www.ncl.ucar.edu/Applications/ESMF.shtml) is used to regird all precipitation products to the SAOBS coordinate based on first-order conservative operation, which preserves the integral of the source precipitation field across the regridding. In addition, data products with sub-daily temporal resolution are aggregated to monthly data before comparison. Finally, all the precipitation products reach consistency in spatiotemporal resolution that aligns with SAOBS observation. All the evaluations are conducted based on the grid element of 0.5° ×  0.5° at monthly time scale ranging from 2001 to 2017.

**Table 1:** Summary of all the precipitation products.

Product	Spatial resolution	Temporal resolution	Spatial coverage	Temporal range
SAOBS (V2)	0.5° × 0.5°	Daily	20°S to 25°N and 80°E to 180°E	01-01-1981 to 31-12-2017
CPC	0.5° × 0.5°	Daily	Global	1979 to present
CHIRPS	0.25° × 0.25°	Daily	50°S to 50°N	1981 to present
ERA5	0.25° × 0.25°	Hourly	Global	1940 to present
IMERG (V06B)	0.1° × 0.1°	Half hourly	60°S to 60°N	01-06-2000 to 01-10-2021
PERSIANN (V1R1)	0.25° × 0.25°	Daily	60°S to 60°N	1982 to present

### 2.3. Study domain and subdomains

**Fig 1:**
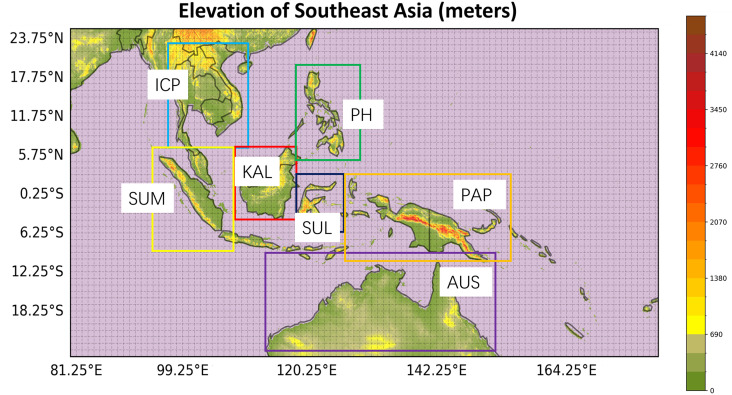
Study region and 7 subdomains of ICP, KAL, SUM, PH, SUL, AUS, and PAP overlaid on the digital elevation model. The map was created using Python package of Cartopy, with digital elevation model sourced from the Shuttle Radar Topography Mission (SRTM).

The domain of SEA is located from 15° S to 35° N and 85°E and 155°E (Fig 1). Due to the spatial and temporal variability of precipitation patterns over SEA, the study area is divided into seven subdomains as suggested in previous studies [34–36]: The Indochina Peninsula (ICP; 6°N to 23°N, 95°E to 110°E), Kalimantan (KAL; 4°S to 6°N, 109°E to 118°E), Sumatra (SUM; 8°S to 6°N, 95°E to 108°E), Philippines (PH; 5°N to 20°N, 118°E to 130°E), Sulawesi (SUL; 6°S to 3°N, 118°E to 126°E), Australia (AUS; 25°S to 10°S, 113°E to 152°E), and Papua New Guinea (PAP; 11°S to 4°N, 127°E to 155°E). The evaluation of different precipitation products is performed across the entire SEA and separately for each subdomain.

### 2.4. Evaluation metrics

For quantitatively evaluating the five precipitation products in terms of population, spatial distribution and seasonal variability, we apply three evaluation metrics: correlation coefficient (CC), root-mean square error (RMSE), and relative difference percentage (RDP):


$$\begin{array}{c} CC=\frac{\sum_{i=1}^{N}\left(Product_{i}-\bar{Product}\right)\left(SAOBS_{i}-\bar{SAOBS}\right)}{\sqrt{\sum_{i=1}^{N}{\left(Product_{i}-\bar{Product}\right)}^{2}\sum_{i=1}^{N}{\left(SAOBS_{i}-\bar{SAOBS}\right)}^{2}}}, \end{array}$$ (1)

$$\begin{array}{c} RMSE=\sqrt{\frac{1}{N}\overset{N}{\underset{i=1}{\sum }}{\left(Product_{i}-SAOBS_{i}\right)}^{2}}, \end{array}$$ (2)

$$\begin{array}{c} RDP=\frac{Product-SAOBS}{SAOBS}\times 100\%, \end{array}$$ (3)

where *Product* represents the precipitation amount from the precipitation product to be estimated, *SAOBS* represents the benchmark observation.

To study the differences between SAOBS and precipitation products, scatter plots of monthly mean daily precipitation comparisons are shown to explore the inter-correlation of the assessed products and benchmark dataset. Their probability density functions (PDFs) and cumulative distribution functions (CDFs) are also compared. Regarding the spatial distribution and seasonal variation, additional analyses are conducted to evaluate how well each product captures the spatial patterns and seasonal cycles of precipitation.

## 3. Results

### 3.1. Comparison in data population

**Fig 2:**
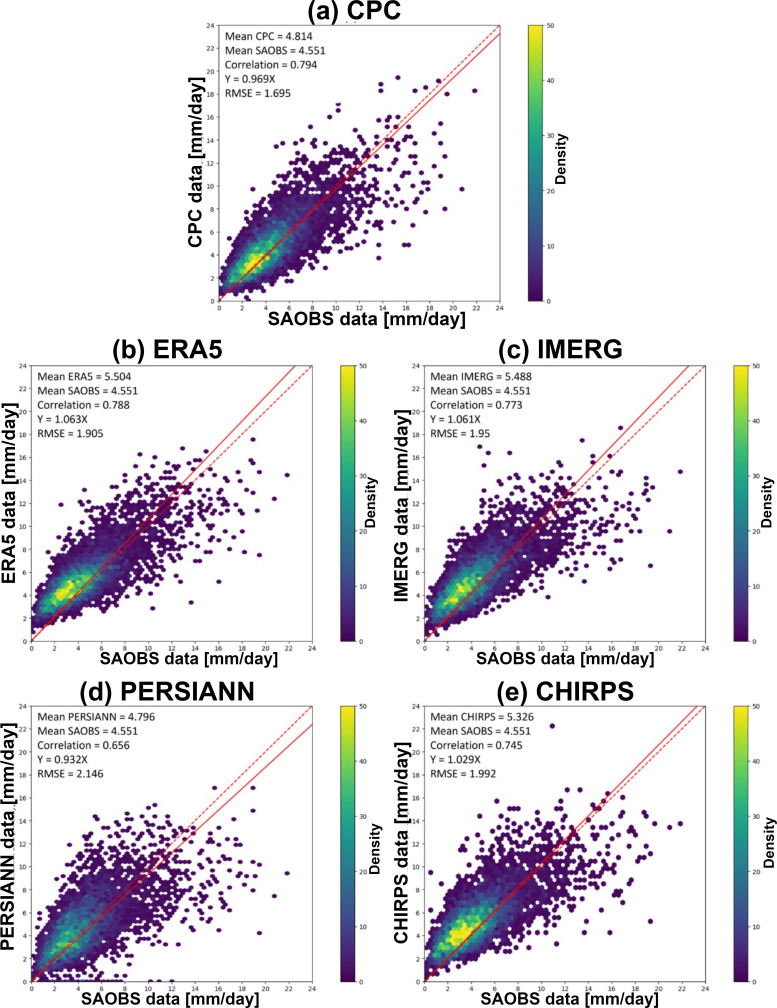
Scatter plot of monthly mean daily precipitation between assessed products and SAOBS. The color bar indicates the number density. (a) CPC, (b) CHIRPS, (c) ERA5, (d) IMERG, and (e) PERSIANN.

To quantitatively assess the overall performance of various products, scatter plots (Fig 2) are used to compare their monthly mean precipitation data for each grid element with SAOBS. In each panel, the dashed line represents the 1:1 reference. The solid line is the linear regression with intercept fixed at 0. The mean precipitation values of both datasets, their CC, regression equation, and RMSE are also included.

All the assessed products, except for PERSIANN, have CC values with SAOBS exceeding 0.74. CPC demonstrates the highest consistency, with a CC value of 0.80. Three products—CHIRPS (1.03), ERA5 (1.06), and IMERG (1.06)—show moderate overestimation compared to SAOBS, while CPC (0.97) and PERSIANN (0.93) exhibit underestimation. In terms of RMSE, CPC performs the best with a value of 1.70, while PERSIANN has the worst performance at 2.15. Based on the scatter plot comparison, CPC aligns most closely with the benchmark, showing the highest CC and lowest RMSE, whereas PERSIANN ranks the lowest. The remaining three products display comparable performance in the middle range.

In terms of the population density of the scatter, all products exhibit a core of dense population between 1 to 6 mm/day, accounting for 70% of the total population. The core region of CHIRPS, ERA5, and IMERG is all skewed above the 1:1 reference, indicating an overestimation of precipitation within this range by the three products. In contrast, the population density of CPC and PERSIANN is symmetrically distributed around the 1:1 reference line, suggesting no systematic bias.

**Fig 3:**
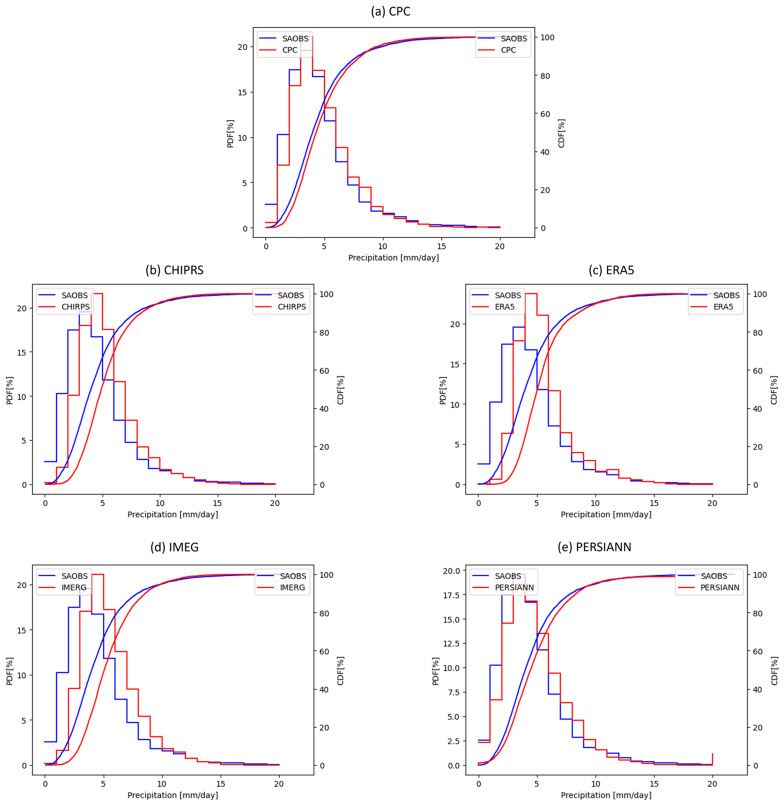
PDFs and CDFs for SAOBS (blue) and the assessed products (red) of monthly mean daily precipitation (mm/day). (a) CPC, (b) CHIRPS, (c) ERA5, (d) IMERG, and (e) PERSIANN.

To directly compare the population distribution of precipitation products with SAOBS, Fig 3 displays the PDFs and CDFs of SAOBS and the five evaluated products. CHIRPS, ERA5, and IMERG show a noticeable horizontal shift towards higher values in their PDFs compared to SAOBS and a clear separation in their CDFs at values below 10 mm/day. These results align with the scatter plot findings, indicating that these three products tend to overestimate daily precipitation intensities below 10 mm/day, which are most common in the study area. In contrast, CPC and PERSIANN do not exhibit such skewness, indicating no systematic bias in these two products.

### 3.2. Comparison in spatial distribution

**Fig 4:**
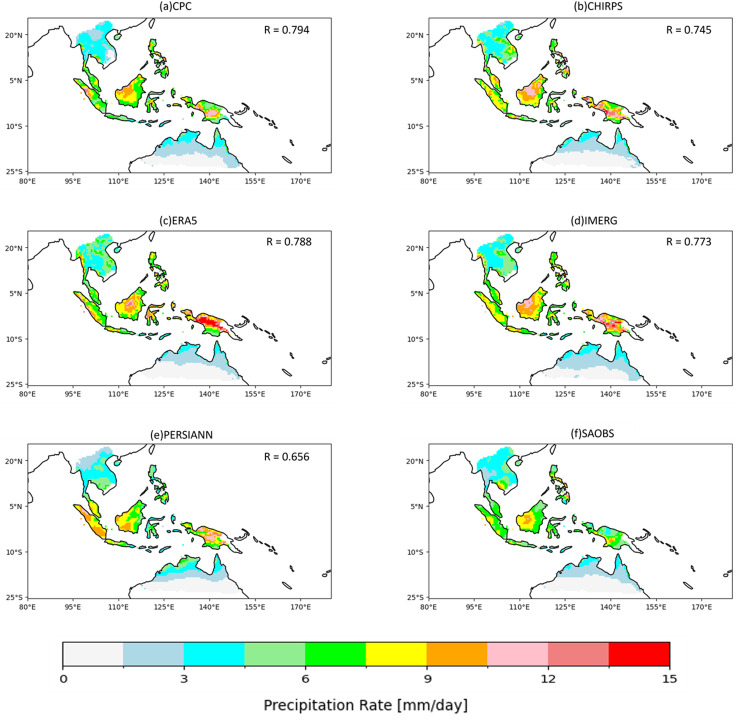
Spatial distribution of annual mean precipitation during the period 2001-2017. (a) CPC, (b) CHIRPS, (c) ERA5, (d) IMERG, (e) PERSIANN, and (f) SAOBS.

All assessed precipitation products (**Fig** 4a-e) show a consistent spatial pattern that featuring the high precipitation close to the equator but the intensity decreases towards higher latitudes, which align well with the benchmark observation (Fig 4f). However, all 5 products demonstrate notable overestimation over the high-precipitation regions relative to SABOS, particularly in the PAP, KAL, and SUM regions, where the observation typically ranges from 5 to 10 mm/day, while the assessed products commonly exceed 12 mm/day, and the overestimation is most prominent for CHIRPS (Fig 4b), ERA5 (Fig 4c), and IMERG (Fig 4d).

**Fig 5:**
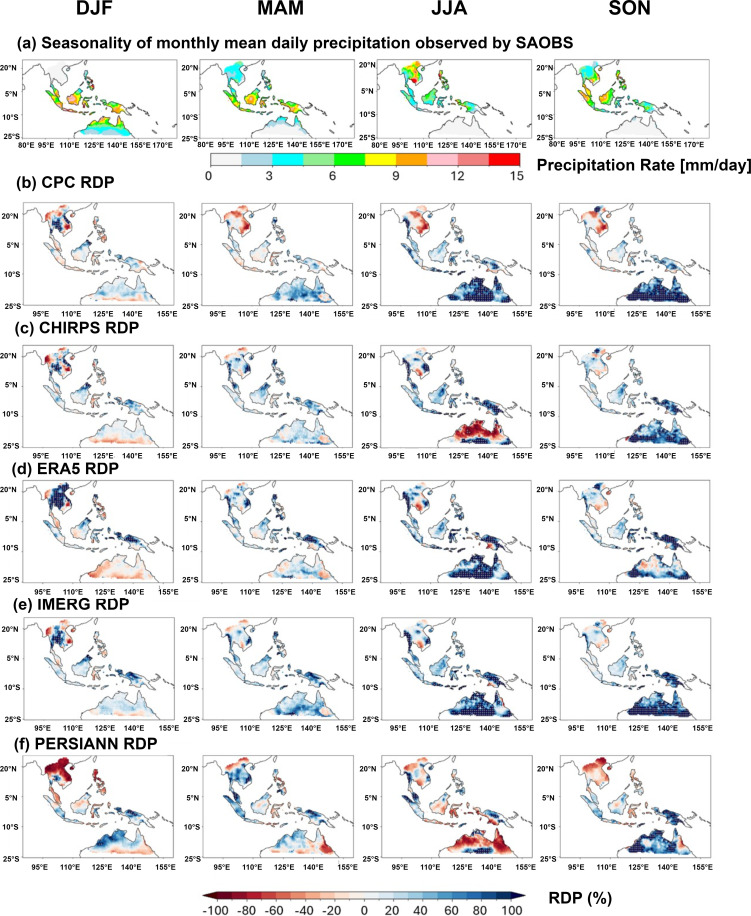
(a) Spatial distribution of seasonal mean daily precipitation from SAOBS observation. The seasonal RDP of the assess products during the period 2001-2017. The gray crosses indicate the extremes (below -100% or above 100%). (b) CPC, (c) CHIRPS, (d) ERA5, (e) IMERG, and (f) PERSIANN [12].

Comparing the spatial distribution of annual mean daily precipitation might overshadow the seasonal performance of the evaluated products. Therefore, a more detailed comparison in seasonal spatial distribution is presented in Fig 5. Fig 5a shows the seasonal mean daily precipitation of SAOBS for December-January-February (DJF), March-April-May (MAM), June-July-August (JJA), and September-October-November (SON). Accordingly, RDP values of each assessed products are calculated with the reference of the benchmark dataset (**Fig** 5b-f).

During the Northeast Asian Monsoon (DJF), the equatorial SEA (SUM, KAL, SUL, and PAP) receives the most abundant rainfall, while it corresponds to the drought season over the continental SEA (i.e., ICP). Such regional contrast is well captured by CPC. However, CPC has a notable drawback over ICP, though it significantly overestimates precipitation in ICP. Compared to CPC, all other products show more prominent overestimation in the equatorial SEA, particularly over PCP and ICP. PERSIANN stands out as it severely underestimates precipitation in ICP, PH, and the Malay Peninsula. Therefore, when applying these precipitation products to study DJF precipitation, caution should be exercised regarding overestimation in ICP and PAP. Among the products, CPC performs the best, while PERSIANN ranks the lowest.

During the intermonsoon season of MAM, ICP transitions from drought to the wet season, while rainfall in AUS decreases. The equatorial SEA remains the wettest region, with CPC still showing the best performance there. However, CPC severely underestimates precipitation in ICP and notably overestimates it in AUS. CHIRPS, ERA5, IMERG, and PERSIANN consistently overestimate precipitation across the entire SEA domain, with particularly notable overestimation in PAP.

When Southwest Asian Monsoon prevails (JJA), the precipitation core shift northward from equatorial SEA to continental SEA, and AUS enters dry season. Over equatorial SEA, CPC outperforms all other products with the minimal overestimation. Similar as MAM, CPC exhibits widespread underestimation in ICP, which is not as optimal as other products. As for AUS, CPC, ERA5, and IMERG all show significant overestimation, whereas CHIRPS and PERSIANN correspond to significant underestimation. During the intermonsoon season of SON, all products perform similarly to their performance in JJA, with consistent overestimation in AUS across all products.

In summary, CPC surpasses all other products in estimating precipitation over equatorial SEA throughout all seasons. However, it consistently underestimates precipitation over continental SEA (ICP) year-round. As for AUS, all products show decent estimation during the wet seasons of DJF and MAM, but stronger deviation is observed during the dry season of JJA and SON.

### 3.3. Comparison in temporal distribution

**Fig 6:**
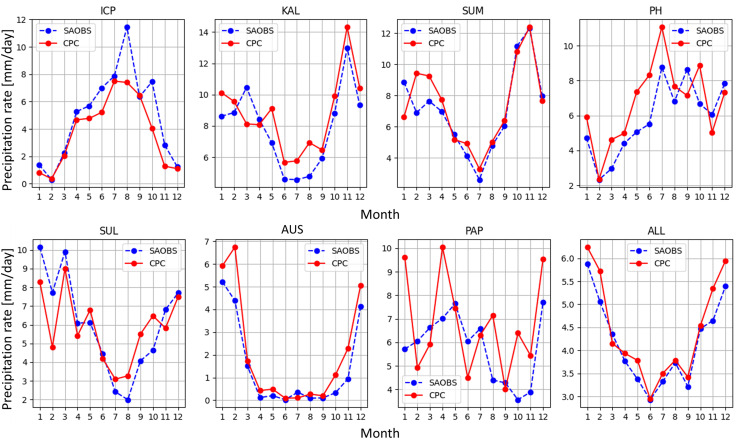
Monthly mean precipitation of CPC (red solid) and SAOBS (blue dashed) for each region.

Fig 6 shows the comparison of monthly mean domain-average daily precipitation between CPC and SAOBS for each month. Except for PAP, where CPC shows dramatic month-to-month changes, the precipitation pattern of CPC matches well with the benchmark dataset. The performance of the rest of the assessed products ( S1-S4 **Fig**) is not as optimal as that of CPC.

**Fig 7:**
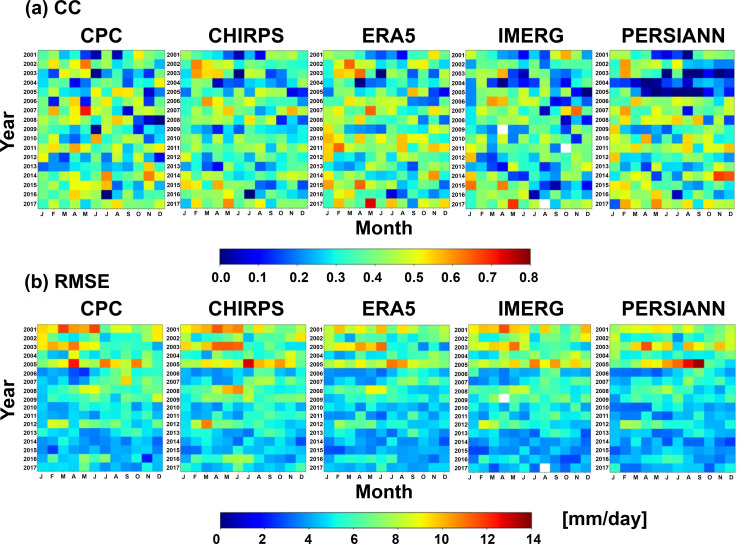
(a) Correlation coefficients and (b) root-mean square errors between SAOBS and the assess products for the monthly sequence of precipitation.

To determine if there is a year-by-year bias, CC and RMSE values are compared between precipitation products and SAOBS (Fig 7). The x-axis represents the month, and the y-axis represents the year. CC values are below 0.8, with an average of about 0.4 to 0.5. Lower correlation values are concentrated between 2001 and 2006. For RMSE, it is evident that from 2001 to 2006, the RMSE is mostly above 9.0 mm/day, while during the remainder of the study period, it is generally below 6 mm/day. These findings indicate a potential bias in the precipitation products, with the years of 2001-2006 exhibiting greater discrepancies between the observed and estimated values. Revealing such is crucial for understanding the accuracy of precipitation estimates and enhancing the reliability of these products for various applications.

## 4. Conclusion and discussion

This study evaluates the performance of precipitation data from 5 products (CPC, CHIRPS, IMERG, ERA5, PERSIANN) over the SEA from January 2001 to December 2017. Based on the comparison between benchmark dataset SAOBS, conclusions are summarized as below.

1. The CPC product consistently outperforms the other products across most metrics and regions, demonstrating the highest correlation coefficient (CC) and the lowest root-mean-square error (RMSE) when compared to the benchmark SAOBS dataset. CHIRPS, ERA5, and IMERG show moderate overestimations, while PERSIANN generally underestimates precipitation.
2. All products show a consistent spatial pattern of high precipitation near the equator, decreasing towards higher latitudes. However, they all overestimate precipitation in high-rainfall regions, particularly in PAP, KAL, and SUM. CPC performs best in equatorial SEA throughout all seasons but underestimates precipitation over the continental SEA year-round.
3. During the Northeast Monsoon (DJF), CPC captures the regional contrast well but overestimates precipitation in ICP. In the intermonsoon season (MAM), CPC underestimates precipitation in ICP and overestimates in AUS. During the Southwest Monsoon (JJA) and the subsequent intermonsoon season (SON), CPC performs better in equatorial SEA but shows significant overestimation in AUS. A potential bias in the temporal sequence is found, with the years of 2001-2006 exhibiting greater discrepancies between the observed and estimated values.

CPC stands out due to its long-term history of refinement and validation, providing consistent and reliable precipitation data over decades—an essential feature for climatological studies. Its reliance on an extensive rain gauge network ensures accurate, localized measurements and reduces biases. In contrast, other datasets have notable limitations: CHIRPS and IMERG heavily depend on satellite data, derived from indirect cloud property measurements rather than direct surface precipitation, which can lead to inaccuracies in regions with complex topography or persistent non-precipitating cloud cover. ERA5, as a reanalysis product, integrates model-driven outputs, which may underperform in areas with limited observational data to constrain the models. PERSIANN, designed for global applicability, prioritizes coverage over regional precision, making it less effective in capturing fine-scale rainfall dynamics. These factors underscore CPC’s robustness, particularly in regions with dense rain gauge networks and variable precipitation patterns.

In conclusion, while each precipitation product has its strengths and weaknesses, CPC emerges as the most reliable for the SEA region, with specific caution needed for its underestimation and overestimation tendencies in different subdomains and seasons. Future research should focus on improving the regional tuning of these products to enhance their accuracy for specific applications in Southeast Asia.

It is important to note that while differences are observed in terms of CC and RMSE, no statistical significance is found after performing the two-tailed t-test (significance level of 0.05) between CPC and the other datasets. This suggests that CPC’s outperformance may result from random variation rather than systematic bias. Furthermore, SAOBS, as a gridded product derived from a limited number of station observations, is subject to substantial uncertainties stemming from data processing, interpolation methods, and the choice of weighting functions. As a result, the evaluation of gridded products against station-level observations is generally preferred [12]. However, in the absence of such station data, further investigation is warranted in follow-up studies.

## Supporting information

S1 Fig Same as Fig 6, but for CHIRPS. (TIF)

S2 Fig Same as Fig 6, but for ERA5. (TIF)

S3 Fig Same as Fig 6, but for IMERG. (TIF)

S4 Fig Same as Fig 6, but for PERSIANN. (TIF)
