# Supplementary figures and images for: Assessing the monthly performance of daily precipitation products over Southeast Asia using the gauge-based analysis
Source: PLoS One. 2025 Mar 25;20(3):e0319477. doi: 10.1371/journal.pone.0319477 (PMC11936298; doi:10.1371/journal.pone.0319477)

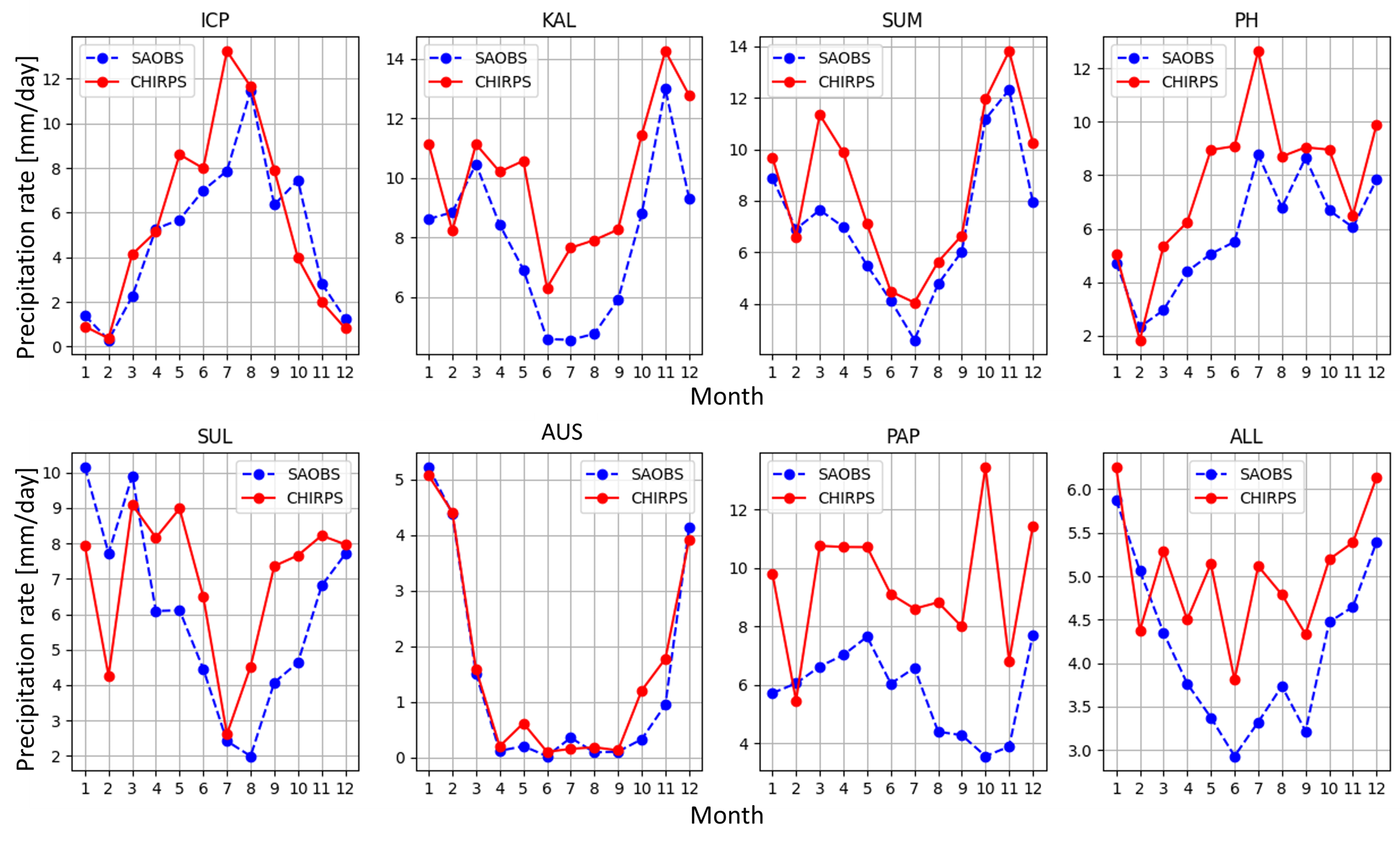

Supplement: S1 Fig — (TIF) [file pone.0319477.s001.tif]

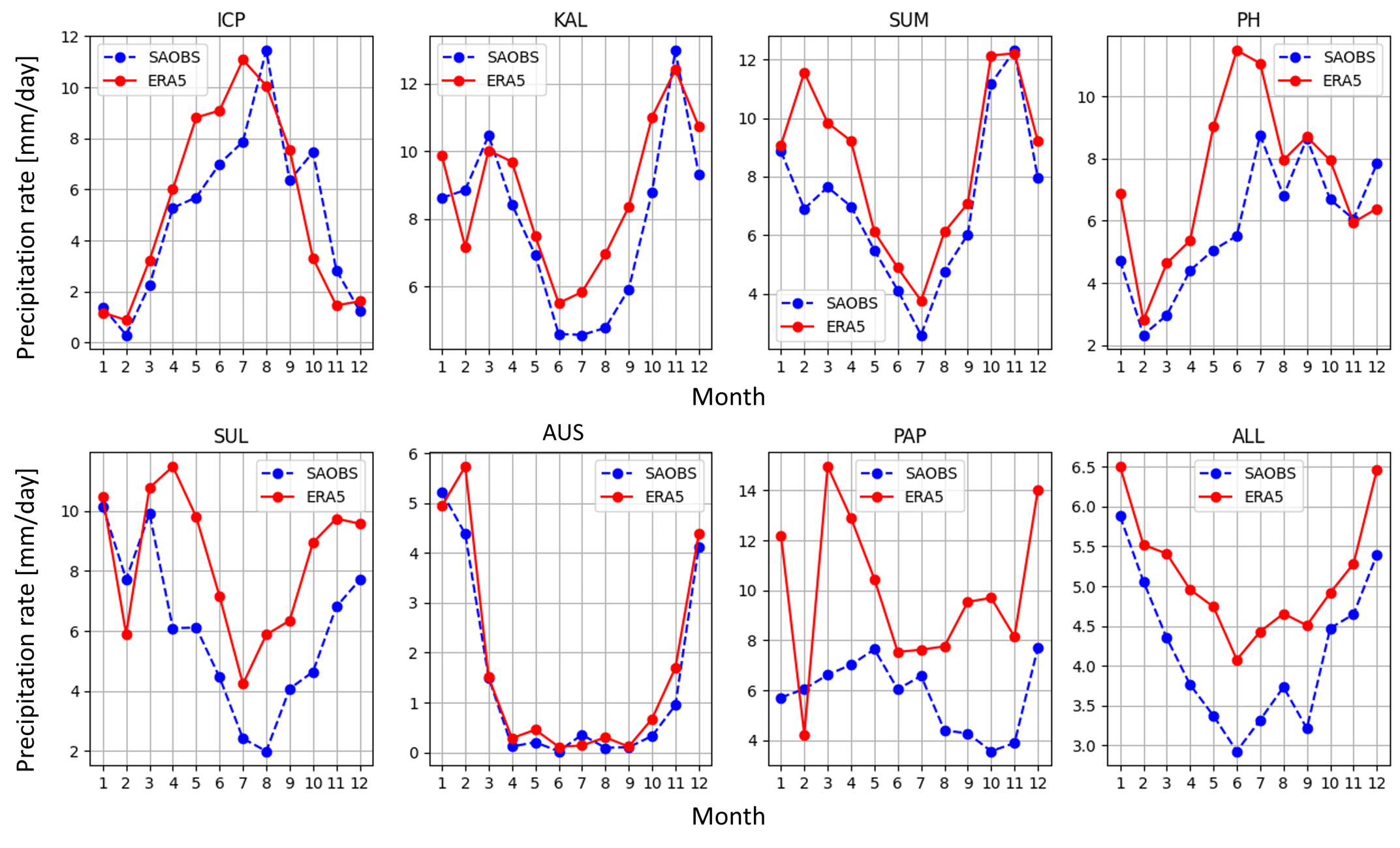

Supplement: S2 Fig — (TIF) [file pone.0319477.s002.tif]

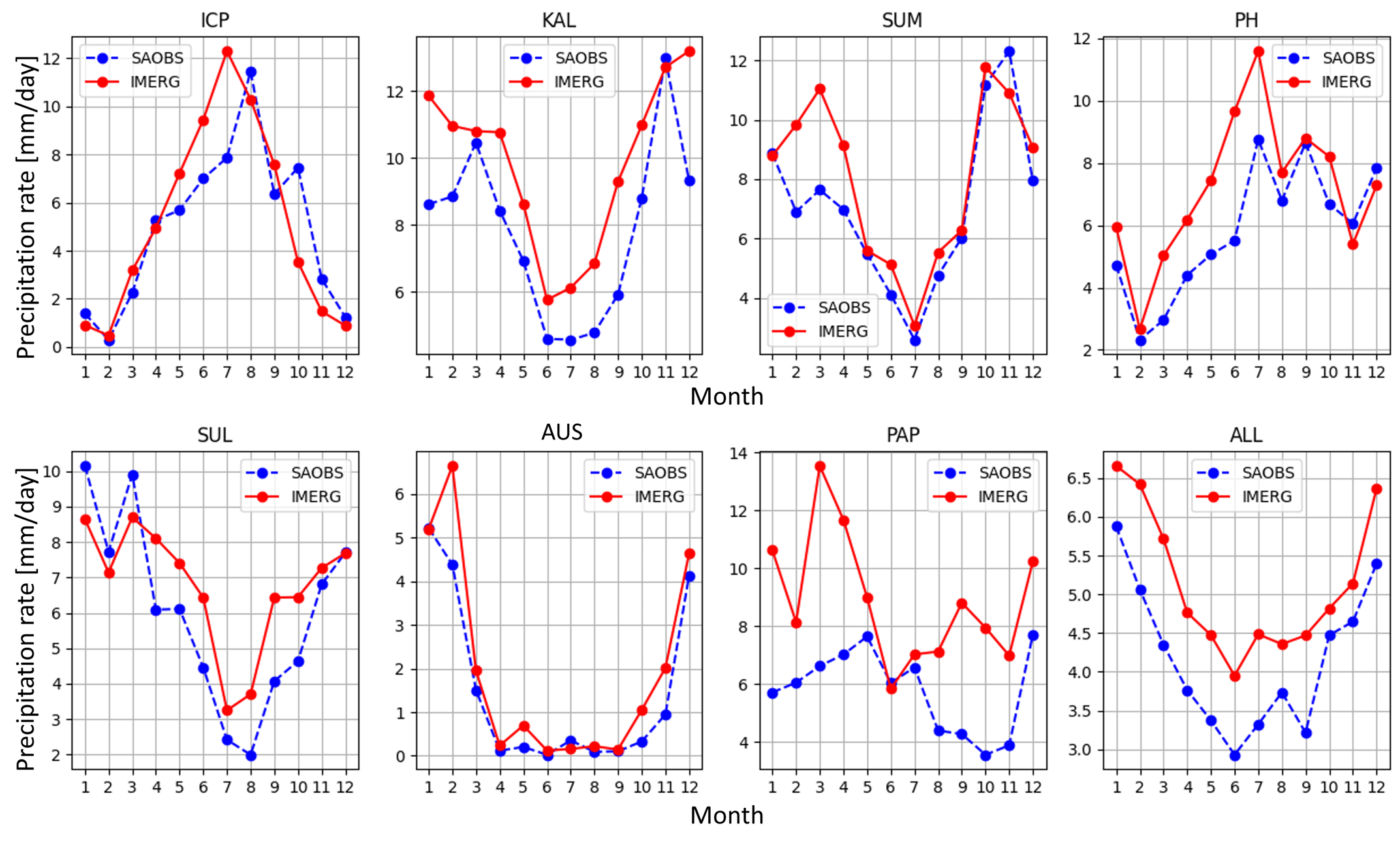

Supplement: S3 Fig — (TIF) [file pone.0319477.s003.tif]

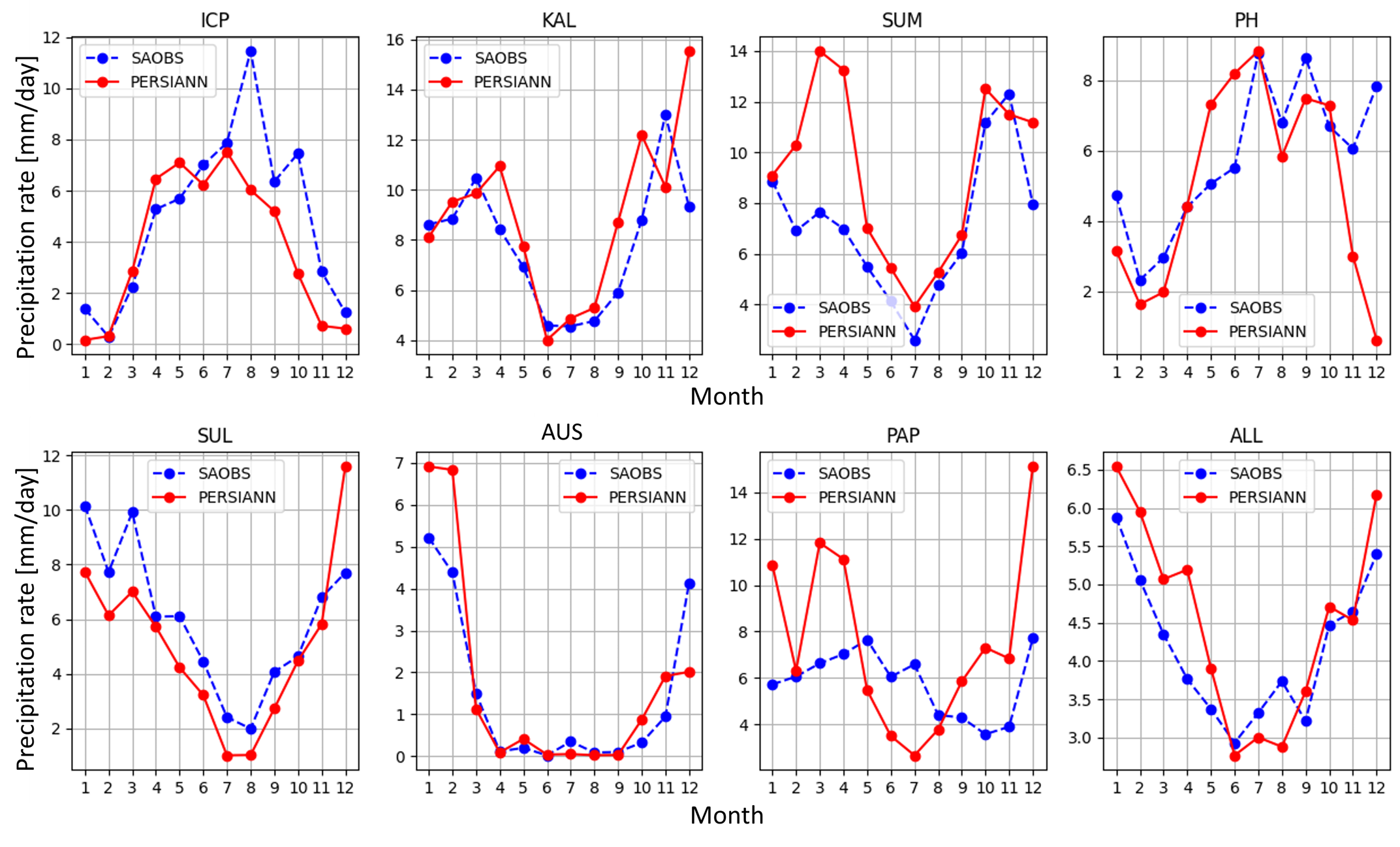

Supplement: S4 Fig — (TIF) [file pone.0319477.s004.tif]
